# Contrast-enhanced US with Perfluorobutane(Sonazoid) used as a surveillance test for Hepatocellular Carcinoma (HCC) in Cirrhosis (SCAN): an exploratory cross-sectional study for a diagnostic trial

**DOI:** 10.1186/s12885-017-3267-8

**Published:** 2017-04-18

**Authors:** Ji Hoon Park, Mi-Suk Park, So Jung Lee, Woo Kyoung Jeong, Jae Young Lee, Min Jung Park, Kyunghwa Han, Chung Mo Nam, Seong Ho Park, Kyoung Ho Lee

**Affiliations:** 1 Department of Radiology, Seoul National University Bundang Hospital, Seoul National University College of Medicine, Institute of Radiation Medicine, Seoul National University Medical Research Center, Gyeonggi-do, Republic of Korea; 2 Department of Radiology and Research Institute of Radiological Science, Severance Hospital, Yonsei University College of Medicine, Seoul, Republic of Korea; 3 Department of Radiology and Research Institute of Radiology, University of Ulsan College of Medicine, Asan Medical Center, Seoul, Republic of Korea; 4 Department of Radiology and Center for Imaging Science, Samsung Medical Center, Sungkyunkwan University School of Medicine, Seoul, Republic of Korea; 5 Department of Radiology and Institute of Radiation Medicine, Seoul National University Hospital, Seoul, Republic of Korea; 6 Department of Radiology, Ajou University Hospital, Ajou University School of Medicine, Suwon, Republic of Korea; 7 Yonsei Biomedical Research Institute, Department of Radiology, Research Institute of Radiological Science Yonsei University College of Medicine, Seoul, Republic of Korea; 8 Department of Biostatistics, Yonsei University College of Medicine, Seoul, Republic of Korea

## Abstract

**Background:**

Ultrasonography (US) is widely used as a standard surveillance tool for patients who are at a high risk of having hepatocellular carcinoma (HCC); however, conventional B-mode US appears to be insufficient in order to ensure the early detection of HCC. Perfluorobutane allows very stable Kupffer phase imaging for at least 60 min, which is tolerable for examinations of the entire liver. The purpose of our study is to evaluate the added value of contrast-enhanced US using perfluorobutane to that of conventional B-mode US as an HCC surveillance tool for patients with liver cirrhosis.

**Methods/Design:**

SCAN (Sonazoid-US for surveillance of hepatoCellulArcarciNoma) is a prospective, multi-institutional, diagnostic trial using an intra-individual comparison design in a single arm of patients. This study was approved by our five institutional review board and informed consent was obtained from all participating. We obtained consent for publication of these data (contrast enhanced US images, CT or MRI images, laboratory findings, age, sex) from all participating patients. All patients will undergo conventional B-mode US immediately followed by contrast-enhanced US. The standardized case report forms will be completed by operating radiologists after B-mode US and contrast-enhanced US, respectively. If any lesion(s) is detected, the likelihood of HCC will be recorded. The primary endpoints are a detection rate of early-stage HCC and a false referral rate of HCC. Intra-individual comparison using Mcnemar’s test will be performed between B-mode US and contrast-enhanced US. The study will include 523 patients under HCC surveillance in five medical institutions in Korea.

**Discussion:**

SCAN is the first study to investigate the efficacy of contrast-enhanced US in surveillance using two reciprocal endpoints specialized for the evaluation of a surveillance test. SCAN will provide evidence regarding whether patients can truly benefit from contrast-enhanced US in terms of the detection of early stage HCC while avoiding additional unnecessary examinations. In addition to the study protocol, we elaborate on potentially debatable components of SCAN, including the design of an intra-individual comparison study, study endpoints, composite reference standards, and indefinite imaging criteria regarding the likelihood of HCC.

**Trial registration:**

The date of trial registration (ClincalTrials.gov: NCT02188901) in this study is July 3, 2014. The last patient enrolled in August 30, 2016 and follow up to see the primary end point is still ongoing. All authors have no other relationships/conditions/circumstances that present a potential conflict of interest of relationships. Our study protocol has undergone peer-review by the funding body (GE Healthcare). No other relationships/conditions/circumstances that present a potential conflict of interest. Also, we clearly stated in the 'competing interests' section of my manuscript.

## Background

Currently, clinical practice guidelines recommend semiannual surveillance for patients who are at a high risk of having hepatocellular carcinoma (HCC) [1–4]. While ultrasonography (US) has been used as a standard examination for HCC surveillance [5], the reported sensitivities, varying from 59% to 92% [6], appear to be insufficient to ensure the early detection of HCC. Using US contrast agents, attempts have been made to improve the efficacy of US in diagnosing HCC; however, US contrast agents in surveillance settings have only rarely been validated [6].

Perfluorobutane (Sonazoid; GE Healthcare, Oslo, Norway) is a second-generation US contrast agent, which has unique characteristics allowing Kupffer phase imaging. Kupffer cells, liver-specific macrophages, phagocytize the perfluorobutane [7] and amplify the ultrasound scattering in order to generate the amplified sound wave. Therefore, a hepatic lesion in which the number of Kupffer cells are either markedly decreased or absent shows a defect on US during the Kupffer phase. Perfluorobutane has been studied for various applications such as the diagnosis of focal liver lesions [8–13], grading of the histologic differentiation of HCCs [14, 15], and guidance of surgical or radiological interventions [16–19].

As the Kupffer phase lasts at least 60 minutes with high stability and allows sufficient time to examine the entire liver, we have noted the value of perfluorobutane as a surveillance tool. One earlier, brief report showed that there was improvement in the detection of small HCCs using perfluorobutane [9]. However, it is still unclear whether patients truly benefit from contrast-enhanced US in terms of the detection of early-stage HCC while avoiding unnecessary additional examinations. The purpose of this study is to investigate the added value of contrast-enhanced US to conventional B-mode US as an HCC surveillance tool in patients with liver cirrhosis.

## Method/design

### Design

Sonazoid-US for surveillance of hepatoCellulArcarciNoma (SCAN) is a prospective, multi-institution, diagnostic trial using an intra-individual comparison design in a single-arm of patients. The primary endpoints are a detection rate of early-stage HCC and a false referral rate. Our study will include 523patientsunder HCC surveillance at five medical institutions (Seoul National University Bundang Hospital, Seoul National University College of Medicine, Severance Hospital, Yonsei University College of Medicine, University of Ulsan College of Medicine, Asan Medical Center, Samsung Medical Center, Sungkyunkwan University School of Medicine, Seoul National University Hospital) in Korea. The institutions were chosen based on their potential to recruit a high number of patients currently in HCC surveillance. The SCAN protocol and the informed consent form have been approved by the ethics committee of each participating institution. Recruitment commenced in October 2014. The date of trial registration (ClincalTrials.gov: NCT02188901) in this study is July 3, 2014. The last patient enrolled in August 30, 2016 and follow up to see the primary end point is still ongoing. All authors have no other relationships/conditions/circumstances that present a potential conflict of interest of relationships.

### Eligibility criteria

Patients aged 20 to 80 years, having liver cirrhosis related to HBV, HCV or primary biliary cirrhosis, and undergoing US for HCC surveillance are eligible for the study. Liver cirrhosis is defined as having one of following criteria [20]: (a) histologically proven liver cirrhosis (METAVIR score 4); (b) endoscopically or radiologically identified esophageal or gastric varices; (c) hepatic surface nodularity seen on a previous cross-sectional imaging study such as US, computed tomography or magnetic resonance imaging; (d)serum platelet count <100,000 /mm^3^; (e) serum albumin <3.5 g/dL; and (f) prothrombin time–international normalized ratio (PT-INR)>1.3. Patients are not eligible for the study if they have history of HCC (either treated or not treated) or a contraindication for the perfluorobutane.

### US technique

**Fig. 1:**
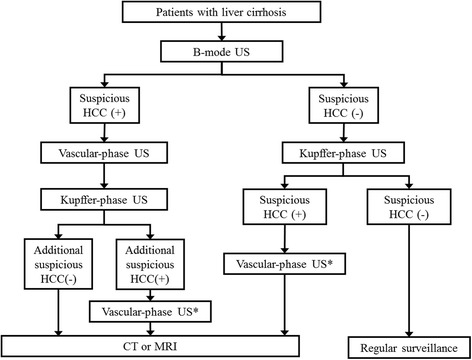
Study flow diagram*After re-administration of perfluorobutane.*CT* computed tomography, *HCC* hepatocellular carcinoma, *MRI* magnetic resonance imaging, *US* ultrasonography

All patients will undergo contrast-enhanced US (Kupffer-phase US ± vascular-phase US) immediately following conventional B-mode US lying in the supine and/or left lateral decubitus position. One of the fellowship-trained abdominal radiologists in each participating institution, and who is aware of the patient’s previous clinical and radiological information, performs B-mode US using an ultrasound system (LOGIQ E9, GE Healthcare, Milwaukee, WI, USA) equipped with a convex transducer operating at a frequency of 1 to 6 megahertz. 16 μg of perfluorobutane is dissolved in 2 mL of sterile water, and the solution is intravenously administrated as a bolus at a dose of 0.015 mL/kg body weight, immediately followed by a 10-mL normal saline flush via the antecubital vein. Ten to 15 minutes after the perfluorobutane administration, the same radiologist performs contrast-enhanced US during the Kupffer-phase with the same ultrasound system. If a new lesion is detected on the preceding B-mode US, vascular-phase US will be performed for the target lesion before the Kupffer- phase and during the arterial (10-40 seconds after administration of perfluorobutane), portal venous (60-90 seconds), and delay (3 minutes)phases. If a new lesion is detected only o n Kupffer-phase US, vascular-phase US will be performed for detecting the target lesion after the Kupffer- phase with re-administration of the same dose of perfluorobutane [9] (Fig. 1). All phases are to be recorded as movie clips. Some representative images, especially in the arterial and Kupffer- phases, are obtained and sent to a picture archiving and communications system (PACS).If there are two or more lesions in a single patient, vascular-phase US will be performed for the largest lesion. In performing US, the radiologists follow the guidelines of the Korean Society of Ultrasound in Medicine [21]. Otherwise, there are no specific restrictions regarding the US technique or time constraint. The scanning parameters are detailed in Table 1.

**Table 1:** Contrast-enhanced US performed according to theperfluorobutane(Sonazoid) protocol

Imaging protocol	
Intravenous access	Antecubital or forearm
Contrast preparation	16 μg of perfluorobutane dissolved in 2 mL of sterile water
Injection dose	0.015 mL/kg body weight
Injection method	Bolus with 10-mL normal saline flush
Contrast US setting	
Scanning view	Dual view ( B-mode + contrast mode)
Mechanical index	0.20 -0.26
Dynamic range	60 - 65dB
Location of the beam focus	Posterior margin of the liver
Contrast harmonic imaging	Used
Kupffer phase	10 to 15 minutes after the injection of perfluorobutane
Vascular phase	After reinjection of perfluorobutane
Artery phase	10-40 seconds
Portal phase	60-90 seconds
Delay phase	3–4 minutes

### Image interpretation

The standardized case report forms will be completed by the operating radiologist after completion of B-mode US and contrast-enhanced US respectively, and will include the subjective quality of the US examination (acceptable or incomplete), the presence or absence of a lesion, and the presence of portal-venous thrombosis. If any lesion(s) is detected, the number, size, location according to Couinaud’s classification, sonographic features, enhancement pattern, and the likelihood of HCC (using a 3-point Likert scale: benign, probably benign, and suspicious HCC) of the lesion(s) are also to be recorded. The imaging criteria for the likelihood of HCC are not protocolled, and the judgement for the likelihood of HCC will be left to the discretion of the radiologists.

### Further imaging work-up

If a patient has any lesion(s) of which the likelihood of HCC is grade 3 (suspicious HCC) on either B-mode US or contrast-enhanced US, the patient will undergo CT or MRI for further characterization of the lesion(s) within 60 days after the surveillance US. For patients in whom no lesion is identified or in those with a lesion in which there is a likelihood of HCC ≤ grade 2 on both B-mode US and contrast-enhanced US, follow-up imaging will be recommended according to the standard surveillance program of each participating institution.

### Reference standards

For patients undergoing hepatic surgery or biopsy, the final diagnosis will be determined based on the pathological HCC criteria as follows [22]. (a) The hepatocellular origin of the tumor verified by architectural and cytological evidence of hepatocellular differentiation. (b) The presence of malignant features such as nuclear atypia and architectural alteration. Three immunohistochemical markers including glypican 3, heat shock protein 70, and glutamine synthetase are used for differentiating between the high-grade dysplastic nodule and well-differentiated HCC. If tumor cells are positive for two or three markers, the tumor will be diagnosed as well-differentiated HCC. For patients who do not undergo surgery or biopsy, the radiology expert panel in the central review unit will determine the final diagnosis using CT or MRI based on the Liver Imaging Reporting and Data System (LI-RADS) version 2014 [23, 24]. Image findings compatible with LR-4, LR-5 and LR5V will be diagnosed as HCC. The expert panel consists of radiologists who are not involved in the prospective US examination. They will be aware of the study concept and design, although they will be blinded to the details of the US results. As all analyses in SCAN will be performed in a per-patient manner, there will be no additional process which determines if a lesion identified on CT or MRI matches the lesion identified on the surveillance US.

### Primary endpoints

The primary study endpoints are a detection rate of early-stage HCC and a false referral rate. The detection rate of early-stage HCC is defined as the proportion of patients having both positive US results and confirmed as having early-stage HCCs on reference standard procedures out of all patients enrolled in the study. The early-stage HCC is defined as a single HCC<5cm or up to three HCCs, each of which is less than 3 cm based on the Barcelona Clinic Liver Cancer staging system (BCLC stage 0 or A) [25]. The false referral rate is defined as the proportion of patients having positive US results but confirmed as not having HCC on reference standard procedures out of all patients enrolled in the study.

The likelihood of HCC = grade 3 will be regarded as test positive both for B-mode US and contrast-enhanced US. If a patient has two or more lesions, the lesion assigned the greatest likelihood of HCC will be regarded as the representative lesion of that patient. As patients who have positive results either on B-mode US or on contrast-enhanced US subsequently undergo reference standard procedures, the true-positives and false referrals are to be determined by comparing the case report forms of each US method with the reference standards. For example, if a patient has a lesion of which the likelihood of HCC is grade 3 on B-mode US but is grade 2 on contrast-enhanced US, and if the patient is finally confirmed as not having HCC on reference standard procedures, it will be counted as a false referral of B-mode US but not of contrast-enhanced US.

### Secondary endpoints

The secondary endpoints include a detection rate of HCC of any stage adverse event rate of perfluorobutane. The detection rate of HCC of any stage is defined as the proportion of patients having both positive US results and confirmed HCCs of any stage out of the patients enrolled in the study. The adverse event rate is defined as the proportion of patients having an adverse event following injection of perfluorobutane out of the patients enrolled in the study. Short-term and long-term adverse events are to be recorded according to the Common Terminology Criteria for Adverse Events (CTCAE version 4.0) [26] with structured telephone interviews three and 90 days after the surveillance US.

### Stopping rule

If one or more life-threatening or fatal serious adverse events (SAEs) are reported in any of study patients, SCAN will be suspended and the study coordinating committee will investigate if the events are attributable to study procedures and determine whether or not SCAN should be terminated early.

### Sample size

For sample size calculation, we assumed a 5% prevalence of HCC in our target population, a 3.15% detection rate of early-stage HCC on B-mode US (63% of sensitivity)[27],and a4.75% detection rate of early-stage HCC on contrast-enhanced US (95% of sensitivity) [8]. We also assumed a 1.7% discordant rate which included0.05% of the proportion of patients in whom a lesion is detected on B-mode US but not on contrast-enhanced US. With these assumptions, 523patients are needed to obtain 80% statistical power for the McNemar’s test with an α equal to 0.05.

### Data management

Trained research associates will perform data checks for accuracy and completeness. Using an electronic case report form, the data will be entered into the database. The collected data will be kept in the central data archive of the data center, which has a built-in security feature preventing unauthorized access to confidential participant information.

### Data analysis

While intention-to-treat analysis will be primarily performed for all patients enrolled in the study, per-protocol analysis will also be performed for patients who follow the study protocol without violation. Intra-individual comparison will be made between B-mode US and contrast-enhanced US in terms of the detection rate of early-stage HCC, the false referral rate, and the detection rate of any stage HCC using McNemar’s test in a per-patient manner.

Subgroup analyses will be performed for each endpoint according to a patient’s age, gender, body mass index, cirrhosis etiology, history of anti-viral treatment, Child-Pugh score, Albumin-Bilirubin (ALBI) grade [28], and alpha-fetoprotein (AFP). We will use logistic regression models with a generalized linear mixed model (GLMM) including random intercept for the patients if the study results show considerable variation across the participating institutions. *P* values less than 0.05 are considered statistically significant. All statistical analyses will be performed using commercially available software (SAS Ver. 9.2; SAS Institute, Cary, NC, USA).

## Discussion

### Study design

We designed SCAN as an intra-individual comparison study rather than a randomized controlled trial (RCT). Due to the lack of previous researcher grading contrast-enhanced US in surveillance, it was impossible to set the appropriate effect size for an RCT. The lack of baseline data also raised a concern regarding the equipoise that could justify the randomization. Although we could not use the design of an RCT, intra-individual comparison has obvious advantages as it would allow for an excellent comparability between a pair of results and would also increase the statistical power for a given number of study patients. Unlike typical therapeutic or interventional trials, the diagnostic examinations investigated in diagnostic trials can be repeatedly applied to the same patient without affecting the disease status. We expect that the results of SCAN will offer not only the efficacy of contrast-enhanced US but also relevant baseline data for future studies on US surveillance.

### Study endpoints

The accuracy of a diagnostic test can ideally be evaluated with a definitive diagnosis, usually in terms of sensitivity and specificity. However, studies investigating the efficacy of US in surveillance settings have frequently been limited by verification bias [27]. This is because the definitive diagnostic procedure is used on patients who have positive results, while those who have negative results are not confirmed as true or false negatives, which indicates that neither sensitivity nor specificity is identifiable [29]. A detection rate and a false referral rate are two important reciprocal measures which do not require definitive diagnoses from all study patients and allow a comparison of the relative performance of two screening tests [29, 30]. While we expect improvement of the detection rate of early-stage HCC by adding contrast-enhanced US to conventional B-mode US, the improvement can be truly valuable only if the false referral rate is decreased or at least remains stable.

### Composite reference standards

We set composite reference standards in SCAN, which uses either histopathology or radiology as the final diagnosis of HCC. Various treatments, such as surgery, radiofrequency ablation, percutaneous ethanol injection, and combined therapy of radiofrequency ablation and trans-arterial chemoembolization, are currently available for early-stage HCCs [1, 2, 31]. This makes the investigation of the efficacy of a diagnostic test for HCC more complex as histopathology, which has traditionally been used as the reference standard for imaging diagnosis, is not available in most patients. Furthermore, the very high specificity of the typical radiological features of HCC in an at-risk population rendered the imaging diagnosis a substitute for histopathology, and the recently updated American Association for the Study of Liver Diseases (AASLD) guidelines have even proposed that one imaging technique (either CT or MRI) showing the typical radiological features suffices for diagnosing HCC [2]. It is now regarded as unethical to perform a biopsy on a patient with HCC, in whom the biopsy is unnecessary for clinical purposes. If we use only histopathology as the reference standard, it should result in a significant bias as then only HCCs showing atypical radiological features will be included as true HCCs.

### Imaging criteria for the likelihood of HCC

As the imaging criteria for the likelihood of HCC are not protocolled in SCAN, the diagnosis will be left to the discretion of the radiologists who conduct and interpret the US. In previous research studies evaluating the efficacy of US in the surveillance setting, the criteria defining which lesion should trigger further investigation were usually absent or, if any, they differed from the other criteria [32–43]. Considering the subjectivity of US interpretation and the diverse sonographic features of HCC [44], the current guidelines often suggest that any new lesion detected on US, regardless of its echogenic pattern, should be further evaluated by CT or MRI. However, there is still a discrepancy between the guidelines regarding the use of the size criterion. While AASLD and The European Association for the Study of the Liver (EASL)guidelines suggest a 1-cm criterion [1, 2], guidelines from the Asian-Pacific region do not have such a criterion in the diagnostic algorithms [3, 45, 46]. Furthermore, the clinical significance of a lesion smaller than 1 cm and showing a defect on Kupffer-phase US has not yet been established. Therefore, we have decided not to list specific imaging criteria for the likelihood of HCC which do not reflect current variations in clinical practice.

SCAN is the first study to investigate the efficacy of contrast-enhanced US in surveillance using two reciprocal endpoints specialized for evaluation of a surveillance test. SCAN will provide evidence regarding whether patients can truly benefit from contrast-enhanced US in terms of the detection of early stage HCC while avoiding additional unnecessary examinations.

## Abbreviations

AFP: Alpha-fetoprotein
ALBI: Albumin-Bilirubin
BCLC: Barcelona Clinic Liver Cancer staging system
CTCAE: Common Terminology Criteria for Adverse Events
EASL: The European Association for the Study of the Liver
GLMM: Generalized linear mixed model
HCC: Hepatocellular carcinoma
LI-RADS: Liver Imaging Reporting and Data System
PACS: Picture archiving and communications system
RCT: Randomized controlled trial
SAEs: Serious adverse events
US: Ultrasonography
